# Oral Valganciclovir Therapy in Infants Aged ≤2 Months with Congenital Cytomegalovirus Disease: A Multicenter, Single-Arm, Open-Label Clinical Trial in Japan

**DOI:** 10.3390/jcm11133582

**Published:** 2022-06-21

**Authors:** Ichiro Morioka, Yasumasa Kakei, Takashi Omori, Kandai Nozu, Kazumichi Fujioka, Naoto Takahashi, Tetsushi Yoshikawa, Hiroyuki Moriuchi, Yoshinori Ito, Akira Oka

**Affiliations:** 1 Department of Pediatrics and Child Health, Nihon University School of Medicine, Tokyo 173-8610, Japan; ito.yoshinori@nihon-u.ac.jp; 2 Department of Oral and Maxillofacial Surgery, Kobe University Graduate School of Medicine, Kobe 650-0017, Japan; ykakei@med.kobe-u.ac.jp; 3 Clinical and Translational Research Center, Kobe University Hospital, Kobe 650-0017, Japan; 4 Division of Biostatistics, Department of Social/Community Medicine and Health Science, Kobe University Graduate School of Medicine, Kobe 650-0017, Japan; tomori@med.kobe-u.ac.jp; 5 Department of Pediatrics, Kobe University Graduate School of Medicine, Kobe 650-0017, Japan; nozu@med.kobe-u.ac.jp (K.N.); fujiokak@med.kobe-u.ac.jp (K.F.); 6 Department of Pediatrics, The University of Tokyo, Tokyo 113-8655, Japan; ntakahashi0819@g.ecc.u-tokyo.ac.jp (N.T.); oka.akira@saitama-pho.jp (A.O.); 7 Department of Pediatrics, Fujita Health University School of Medicine, Toyoake 470-1192, Japan; tetsushi@fujita-hu.ac.jp; 8 Department of Pediatrics, Nagasaki University Graduate School of Biomedical Sciences, Nagasaki 852-8501, Japan; hiromori@nagasaki-u.ac.jp; 9 Department of Pediatrics, Nagoya University Graduate School of Medicine, Nagoya 466-8560, Japan; 10 Saitama Prefectural Children’s Medical Center, Saitama 330-8777, Japan

**Keywords:** auditory brainstem response, clinical trial, cytomegalovirus, neonate, neutropenia, valganciclovir

## Abstract

Our aims were to determine the clinical impact of oral valganciclovir (VGCV) in infants aged ≤2 months with congenital cytomegalovirus (CMV) disease and evaluate the efficacy of VGCV when initiated beyond the neonatal period. The multicenter, single-arm, open-label clinical trial was conducted in Japan. Twenty-five infants aged ≤2 months with congenital CMV disease involving the central nervous system were enrolled and treated with VGCV for 6 months. The primary endpoint was the change in the whole blood CMV load before and after treatment. The secondary endpoint was the change in the auditory brainstem response (ABR) before and after treatment. Changes in ABR were assessed between the younger and older age groups (≤ and >30 days at treatment initiation). Of the 25 patients, one was excluded owing to epilepsy before VGCV administration. The median change in the CMV DNA level in whole blood was −246.0 IU/mL. The best ear and total ear assessments based on ABR were categorized as (improved + unchanged) after treatment for 100% and 93.8%, respectively. No differences in hearing efficacy were observed between the younger and older age groups. Oral VGCV is a potential therapeutic option for treating infants aged ≤2 months with congenital CMV disease.

## 1. Introduction

Congenital cytomegalovirus (CMV) disease, particularly in association with symptomatic infection at birth, is a major cause of neurological sequelae, such as hearing difficulty, epilepsy, neurodevelopmental disorders, and developmental delay [1,2]. We previously conducted an epidemiological survey in Japan that revealed that congenital CMV infection occurs in 1 in 300 births (0.3%) [3]. A Japanese clinical study report showed that “symptomatic” congenital CMV infections, including only with brain image abnormalities, develop in 0.094% of infants born annually, and it is estimated that ~30% of congenital CMV infections are symptomatic [3], which highlights the substantial disease burden in Japanese children.

Earlier clinical trials conducted on newborns within 30 days of age with symptomatic congenital CMV disease involving the central nervous system (CNS) found that hearing difficulty and developmental delay were improved when intravenous ganciclovir (GCV) was administered for 6 weeks and oral valganciclovir (VGCV), which is a prodrug of GCV, for 6 months [4,5,6,7]. Based on such evidence, “A European Expert Consensus Statement” by the European Society for Pediatric Infectious Diseases recommended that a 6-month course of oral VGCV treatment should be the first-line drug for symptomatic congenital CMV disease with associated CNS symptoms or hearing difficulty, and a 6-week to 6-month course for other severe congenital CMV diseases [8]. The International Congenital Cytomegalovirus Recommendations Group also recommended treatments with oral VGCV for moderate to severe symptomatic congenital CMV disease [9]. The oral VGCV treatment is recommended to begin within the first month of life [8,9]. The efficacy of oral VGCV for hearing or psychomotor development and its safety in treatment for 6 weeks or 6 months has been reported in Japan [10,11,12,13,14,15] and globally [16,17,18,19,20,21]. However, VGCV remains unapproved by the government health insurance for symptomatic congenital CMV disease, both in Japan and worldwide. In addition, there is no evidence supporting the efficacy of VGCV when initiated beyond the neonatal period.

A limited number of well-conducted clinical trials investigating treatment options for symptomatic congenital CMV disease have been conducted [7]. Therefore, to facilitate approval by the Japanese government health insurance, we conducted a phase III, investigator-initiated, multicenter, open-label, single-arm clinical trial to evaluate the clinical impact of administering oral VGCV treatment for 6 months to infants aged ≤2 months with congenital CMV disease involving the CNS [22]. This clinical trial aimed to characterize the clinical impact of oral VGCV treatment in infants with symptomatic congenital CMV disease and demonstrate the efficacy of VGCV when initiated beyond the neonatal period.

## 2. Patients and Methods

### 2.1. Study Design and Procedure

Our clinical trial protocol has been previously described [22], and Supplementary Figure S1 provides a summary of the study [22]. The patients enrolled in this clinical trial met all the inclusion criteria (Supplementary Table S1) [22].

Our study recruited 25 participants (including dropouts). This number was calculated based on the proportion of (a) + (b) + (c) for best ear assessment in a previous clinical trial conducted by Kimberlin et al. (for the changes in auditory brainstem response (ABR); four categories were used: (a) improved hearing, (b) no change—normal hearing, (c) no change—same degree of hearing, and (d) hearing deterioration) [5]. Sample size calculation details have been previously outlined [22]. After screening, the participants were orally administered 16 mg/kg VGCV twice daily for 6 months. During the intervention period, the type of feeding, such as breastfeeding and formula milk, was decided at the discretion of the attending pediatrician or neonatologist based on the conditions of the mother and infant.

Supplementary Table S2 presents a summary of the study outcomes, assessments, and procedures [22]. An adverse event was defined as any disease, disability, infection, or death that occurred during the study period [22]. Regarding adverse drug reactions, the criteria for dose adjustment and drug withdrawal were set according to the phase III clinical trial conducted by Kimberlin et al. [7].

This study was conducted in accordance with good clinical practice; the study protocol complied with the Declaration of Helsinki, was approved by the ethics committee of each hospital, and registered in the Japan Registry of Clinical Trials (Identifier: jRCT2051190075) in accordance with recommendations of the International Committee of Medical Journal Editors (registered on 15 November 2019; https://jrct.niph.go.jp/latest-detail/jRCT2051190075) (accessed on 15 June 2022). Written informed consent was obtained from all parents/guardians. The registry was independent of for-profit interests.

### 2.2. Endpoints

The primary endpoint was a change in the whole blood CMV load after treatment for 6 months compared with baseline data. The important secondary endpoint was a change in the ABR (both best ear and total ear hearing assessments) after treatment for 6 months compared with baseline data. Other secondary endpoints included a change in the CMV load dynamics of whole blood and urine [22].

In exploratory endpoints, comparative changes in ABR (best ear and total ear hearing assessment) after treatment for 6 months from baseline values were compared between two groups (younger and older ages divided by 30 days of age at treatment initiation). In 21 infants who completed the treatment for 6 months, changes in ABR (best ear and total ear hearing assessments) were compared between the 6-month and 6-week treatment groups. Adverse events and adverse drug reactions were recorded to assess the safety endpoint.

### 2.3. Statistical Analysis of Endpoints

As demonstrated in our protocol, all analyses were conducted on full data sets obtained from all registered participants who were administered at least one VGCV dose.

For the primary endpoint, the median (minimum and maximum) was calculated for changes in the whole blood CMV load after treatment for 6 months compared with baseline data. The Wilcoxon’s signed-rank test with a significance level of 0.05 (for both sides) was performed under the null hypothesis that the location parameter was 0 for the distribution of change in the whole blood CMV load between levels at the baseline and after treatment for 6 months [22].

For the important secondary endpoint, changes in ABR (both best ear and total ear hearing assessments), four categories were analyzed at baseline and after treatment for 6 months: (a) improved hearing, (b) no change—normal hearing, (c) no change—same degree of hearing, and (d) hearing deterioration [7,22]. The thresholds for hearing were as follows: normal hearing, 0–20 dB; mild hearing abnormality, 21–45 dB; moderate hearing abnormality, 46–70 dB; and severe hearing abnormality, ≥71 dB [5,7]. For the best ear and total ear assessments based on ABR, the proportions and 95% confidence intervals (CI) of (a) + (b) + (c) and (a) + (b) were analyzed by the Wilson score.

For the other secondary endpoints, the following steps were implemented: the dynamics of the CMV load in whole blood and urine during the study period were analyzed, and time plots of the mean with standard deviation (SD) were generated.

For the exploratory endpoints, changes in ABR (both best ear and total ear hearing assessments) between baseline values and after treatment for 6 months were analyzed, and the proportions and 95% CIs of (a) + (b) + (c) and (a) + (b) were calculated and compared between the younger and older age groups using Fisher’s exact test (divided based on the 30 days of age at the start of treatment). In 21 infants who completed the treatment for 6 months, changes in ABR (both best ear and total ear hearing assessments) between baseline values and after treatment for 6 weeks were analyzed, and the proportions and 95% CIs of (a) + (b) + (c) and (a) + (b) were calculated and compared between the 6-month and 6-week treatment groups using the Cochran–Mantel–Haenszel test.

For the safety endpoints, the proportions of adverse events and adverse drug reactions were investigated [22].

## 3. Results

### 3.1. Participant Selection, Baseline Demographics, and Clinical Characteristics

**Figure 1:**
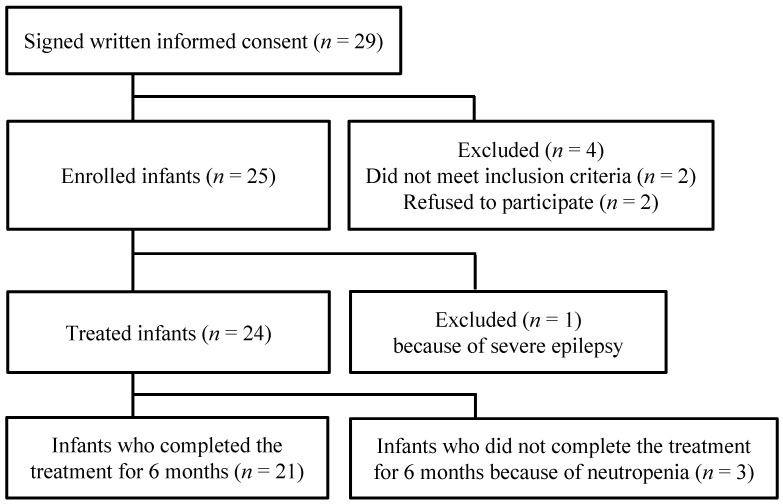
Flowchart showing participant selection for this clinical trial.

The participant selection flow is shown in Figure 1. Written informed consent was obtained from the parents of 29 infants; however, two patients did not meet the selection criteria, and consent was withdrawn for two. Therefore, 25 infants with symptomatic congenital CMV disease were enrolled. One infant was then excluded owing to severe epilepsy caused by intracranial hemorrhage from vitamin K deficiency prior to being administered VGCV medication, and treatment was discontinued for three infants during the trial because of neutropenia. The infants’ baseline demographics and clinical characteristics are summarized in Table 1.

### 3.2. Primary Endpoint

**Figure 2:**
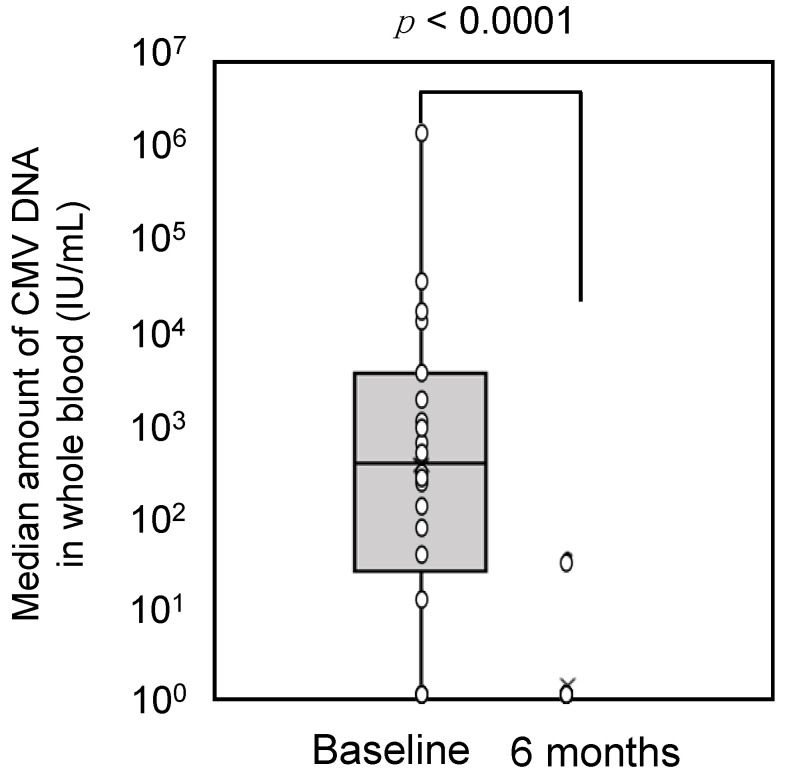
Cytomegalovirus loads in whole blood at baseline and after 6 months of treatment. CMV, cytomegalovirus.

The median change values from baseline of whole blood CMV DNA levels after treatment for 6 months were −246.0 IU/mL (95% CI: −905.0 to −35.0, *p* < 0.0001; Figure 2).

### 3.3. Secondary Endpoints

For the important secondary endpoint, the best ear assessments based on ABR were classified as (improved hearing + no change—normal hearing + no change—same degree of hearing) in 100.0% of the infants and (improved hearing + no change—normal hearing) in 75.0% of the infants (Table 2A). Changes in total ear ABR assessments were (improved hearing + no change—normal hearing + no change—same degree of hearing) in 93.8% and (improved hearing + no change—normal hearing) in 52.1% (Table 2B).

**Figure 3:**
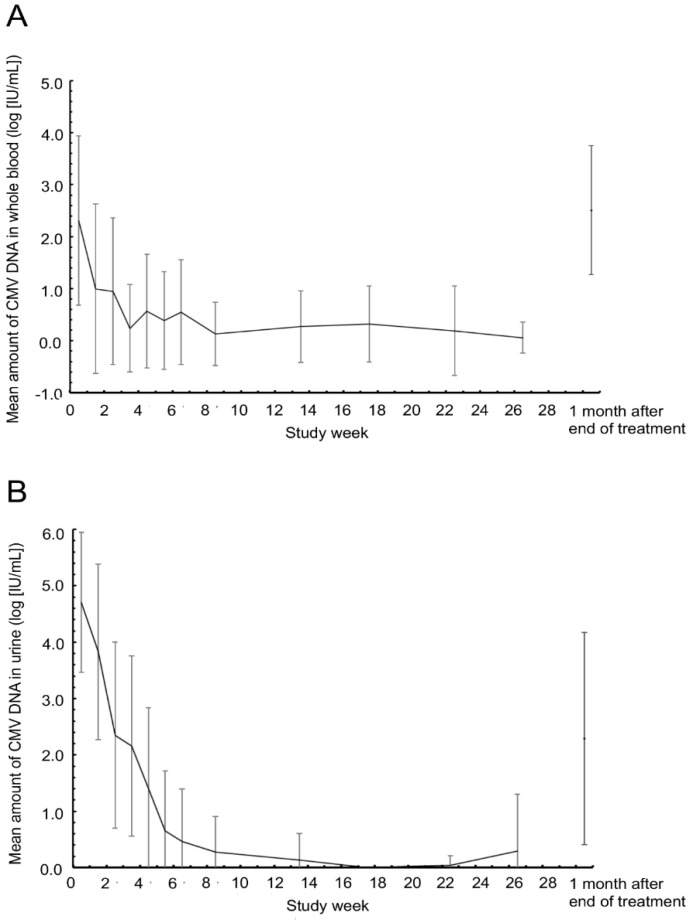
Dynamics of cytomegalovirus load in whole blood (**A**) and urine (**B**) over the study period. CMV, cytomegalovirus.

The mean CMV DNA level (log_10_) in whole blood decreased from 2.31 IU/mL before the start of treatment to 1.00 IU/mL after 1 week and then to 0.24 IU/mL after 3 weeks; it then repeatedly fluctuated and remained below 0.32 IU/mL from 8 to 26 weeks. One month after the end of treatment, the mean CMV DNA level was 2.51 IU/mL (Figure 3A).

The mean urine CMV DNA level (log_10_) gradually decreased from 4.71 IU/mL prior to the start of treatment to below the measurement limit in all infants at 17 weeks. It then subsequently increased to 0.30 IU/mL at 26 weeks and reached 2.29 IU/mL at 1 month after the end of treatment (Figure 3B).

### 3.4. Exploratory Endpoints

When divided into two groups based on the age at the start of treatment (30 days), changes were observed in the best ear hearing assessments based on ABR (improved hearing + no change—normal hearing + no change—same degree of hearing) in 100% of the younger and older age group participants without a significant difference (Table 3A). Changes in the total ear assessment based on ABR were as follows: (improved hearing + no change—normal hearing + no change—same degree of hearing) in 85.7% and 97.1% of participants in the younger and older age groups, respectively, without a significant difference (Table 3B).

In 21 infants who completed the treatment for 6 months, changes in ABR (both best ear and total ear hearing assessments) were compared between the 6-month and 6-week treatment groups. Changes were observed in the best ear hearing assessments based on ABR (improved hearing + no change—normal hearing + no change—same degree of hearing) in 100% of the 6-month and 6-week treatment group participants without a significant difference (Table 4A). Changes in the total ear assessment based on ABR were as follows: (improved hearing + no change—normal hearing + no change—same degree of hearing) in 92.9% and 97.6% of participants in the 6-month and 6-week treatment groups, respectively, without a significant difference (Table 4B).

### 3.5. Safety Evaluations

No infant died. No liver and kidney function abnormalities were observed during the treatment. Adverse events occurred in 19 infants (79.2%). One infant had grade 3 (neutropenia), and eight infants had grade 2 (four cases of neutropenia; one case each of nasopharyngitis, otitis media, and seborrheic eczema; one case of impetigo and diaper dermatitis). Others had grade 1, including five cases of neutropenia. There was a 45.8% incidence (11/24) of adverse drug reactions (10 were neutropenia, and 1 was anemia).

## 4. Discussion

Our clinical trial is a well-conducted study that provides robust evidence regarding the clinical impact and administration safety of oral VGCV in infants aged up to 2 months with symptomatic congenital CMV disease in Japan. Importantly, no significant differences in hearing efficacy were observed between the younger and older age groups.

Regarding the primary endpoint, changes in CMV DNA levels in whole blood significantly decreased between the baseline and 6 months post-treatment. The temporal trend for the CMV DNA levels in whole blood exhibited a rapid decrease from the start of the administration to 4 weeks, which was maintained at a low level thereafter. The same results were observed for the CMV DNA levels in urine. These results indicate that orally administered VGCV is absorbed from the intestinal tract and has an evident viral reduction effect in neonates and infants. VGCV can be reliably used for both initial treatment and maintenance therapy in neonates and infants. This finding is similar to the results of a USA phase III clinical trial [7], suggesting that regardless of race, VGCV is effective for treating neonates and infants with symptomatic congenital CMV disease. This study also confirmed that VGCV can be orally administered as a liquid formulation to neonates and infants.

Hearing difficulties in congenital CMV infection are typically progressive [5,23]. Therefore, it was necessary to determine the clinical impact of VGCV not only for improving hearing but also for maintaining hearing at a particular level. Therefore, one of the secondary endpoints was assessed by “improvement + unchanged (hearing remains normal) + unchanged (same degree of hearing impairment)” regarding the changes in hearing with the best ear. After 6 months of treatment, 100% of patients exhibited improvement or no deterioration. A notable finding was that the best ear assessment revealed that hearing improved in 58.3% of patients. In the assessment of total hearing (both ears), 93.8% exhibited improvements or no changes; although these scores were slightly lower than those of the best ear, they were still considered satisfactory. In contrast, hearing in 3/48 ears (6.3%) deteriorated despite the administration of VGCV. This finding corresponds with that of previous USA phase III clinical trials and our Japanese clinical observational studies, in which corresponding rates of approximately 10% were observed [7,13].

As shown in Figure 3, 1 month after the end of treatment, the viral load of CMV in whole blood and urine increased; this phenomenon had been previously reported [7]. However, this rebound eventually runs its natural course, and the progression of hearing impairment can be suppressed even when a rebound occurs [7]. Therefore, reducing the CMV load during the first 6 months of treatment is paramount.

Intravenous GCV therapy was previously administered for 6 weeks [5]. Thus, we evaluated the administration of VGCV for 6 weeks as an additional exploratory endpoint in this study. There were no apparent significant differences from the baseline in the CMV load in whole blood and the “improvement + no change (hearing remains normal) + no change (same level of hearing impairment)” in the best ear and total ear assessments between the 6-week and 6-month administration periods. The same result was found in the USA phase III clinical trial when evaluated at 12 and 24 months of age; however, the clinical effects on hearing and psychomotor development were significantly higher when treated for 6 months than for 6 weeks [7]. Therefore, to improve the long-term prognosis of affected children, treatment for 6 months is considered superior.

In this clinical trial, we obtained consent within 2 months of birth and started medication within 4 weeks following consent, while in the USA phase III clinical trial, patients were enrolled within 30 days of birth and the drug was administered at an early age (neonatal period) [7]. To verify the effect on hearing following medication administration at an earlier or later age, the patients were evaluated by dividing them into younger and older age groups based on their age (30 days) at the start of drug administration. No significant differences were observed. Therefore, the same effects can be obtained by either administering the drug at an early age (neonatal period) or at 1 to 2 months of age. We consider that this observation is a significant outcome that can be used to inform clinical practice.

Congenital CMV infection occurs not only in patients who show symptoms from birth but also in asymptomatic carriers. Our present clinical trial plan included symptomatic patients with CNS disorders who have a high incidence of developing later disabilities, such as hearing difficulty and developmental delay [1,2]. All 25 patients enrolled (100%) had symptomatic CNS disorders. Because of the nature of the disease, ABR abnormality was commonly observed (87.5%); however, other CNS disorders, such as hydrocephalus and enlarged ventricles (50.0%), white matter disorders (33.3%), and cortical dysplasia (29.2%), were also observed. Among symptomatic congenital CMV infections, patients with a high incidence of later disability were enrolled in our clinical trial.

Regarding safety, grade 3 or 4 neutropenia was reported in approximately 20% of patients in the phase III clinical study conducted in the USA [7]. In our Japanese clinical observational study, neutropenia of 500/mm^3^ occurred in approximately 40% of patients [13]. In this clinical trial, although there was one serious case of grade 3 neutropenia, treatment was discontinued in three patients (12.5%) owing to neutropenia. However, all three patients recovered after discontinuing the study drug. Clinicians should be aware of this potential adverse drug reaction when using oral VGCV therapy, and associated management methods should be provided as a guideline.

As limitations of this clinical trial, first, the recruited patients for our clinical trial were those involving the CNS disorders with positive CMV DNA in urine by an in vitro diagnostic test within 21 days after birth regardless of pre- and perinatal status (Supplementary Table S1). Therefore, the data were not obtained, such as Apgar score and the status of maternal CMV infection. Second, the endpoint for our clinical trial was not included, that is, the efficacy on CNS disorders, in the imaging study after the treatment.

## 5. Conclusions

This is a clinical trial that expounds on the indications of administering oral VGCV to infants with symptomatic congenital CMV disease aged ≤2 months in Japan. Among the study population of 24 patients, VGCV treatment for 6 months reduced the viral load of CMV in whole blood and improved ABR-based hearing test results. In terms of safety, clinicians need to be aware of potential adverse reactions prior to administration. Our results show that oral VGCV is a treatment option for infants aged within 2 months with symptomatic congenital CMV disease in daily practice. Further studies are required to evaluate long-term clinical outcomes.

## Figures and Tables

**Table 1:** Baseline demographic and clinical characteristics, *n* = 24.

**Infants**		
Height at birth (cm)	Mean	45.86
	SD	3.64
	Min.	37.0
	Median	46.85
	Max.	51.0
Body weight at birth (g)	Mean	2367.9
	SD	521.7
	Min.	1304
	Median	2437
	Max.	3306
Gestational age at birth (weeks)	Mean	37.95
	SD	2.18
	Min.	34.0
	Median	38.43
	Max.	41.7
Central nervous system disorder (*n* (%))	Microcephaly	2 (8.3)
	Hydrocephalus or ventricular enlargement	12 (50.0)
	Periventricular calcification	3 (12.5)
	Cortical hypoplasia	7 (29.2)
	White matter injury	8 (33.3)
	Retinal choroiditis	1 (4.2)
	Abnormal auditory brainstem response	21(87.5)
Whole blood CMV load (IU/mL)	Mean	93,739.8
	SD	389,400.6
	Min.	11
	Median	470.0
	Max.	1,701,276
Level of best ear assessment (*n* (%))	Normal	5 (20.8)
	Mild	13 (54.2)
	Moderate	5 (20.8)
	Severe	1 (4.2)
Age at the time of the first dose of medication (days)	Mean	38.2
	SD	13.3
	Min.	14
	Median	35.5
	Max.	66
**Mothers**		
Age (years)	Mean	29.9
	SD	6.2
	Min.	17
	Median	28.5
	Max.	39
Childbirth status (*n* (%))	Primipara	11(45.8)
	Multipara	13 (54.2)
Occupation (*n* (%))	Childcare-giver at nursery	1 (4.2)
	Nurse and health care workers	3 (12.5)
	Others	13 (54.2)
	Unemployed	7 (29.2)

CMV, cytomegalovirus; Max., maximum; Min., minimum; *n*, number; SD, standard deviation.

**Table 2:** Assessments of best ear and total ear hearing using auditory brainstem responses before and after 6 months of treatment.

**A. Best Ear (*n* = 24)**		**Cases (%)**
Change in ABR before and after 6 months of treatment	(a) Improved hearing	14 (58.3)
	(b) No change (normal hearing)	4 (16.7)
	(c) No change (same degree of hearing impairment)	6 (25.0)
	(d) Hearing deteriorated	0 (0.0)
	(a) + (b) + (c)	24 (100.0)
	95% CI ^1^	86.2–100.0
	(a) + (b)	18 (75.0)
	95% CI ^1^	55.1–88.0
**B. Total Ear (*n* = 48)**		**Ears (%)**
Change in ABR before and after 6 months of treatment	(a) Improved hearing	19 (39.6)
	(b) No change (normal hearing)	6 (12.5)
	(c) No change (same degree of hearing impairment)	20 (41.7)
	(d) Hearing deteriorated	3 (6.3)
	(a) + (b) + (c)	45 (93.8)
	95% CI ^2^	87.1–100.0
	(a) + (b)	25 (52.1)
	95% CI ^2^	37.4–66.8

ABR, auditory brainstem response; CI, confidence interval. ^1^ Wilson score. ^2^ Wilson score based on robust standard error considering intraindividual correlation.

**Table 3:** Comparisons of best ear and total ear hearing using auditory brainstem responses after 6 months of treatment, based on the age at the start of treatment (younger vs. older age).

**A. Best Ear**		**Younger Age Group (14–28 Days of Age)**	**Older Age Group (31–66 Days of Age)**	***p*-Value**
		***n* = 7**	***n* = 17**	
		**Cases (%)**	**Cases (%)**	
Change in ABR before and after 6 months of treatment	(a) Improved hearing	4 (57.1)	10 (58.8)	
	(b) No change (normal hearing)	1 (14.3)	3 (17.6)	
	(c) No change (same degree of hearing impairment)	2 (28.6)	4 (23.5)	
	(d) Hearing deteriorated	0 (0.0)	0 (0.0)	
	(a) + (b) + (c)	7 (100.0)	17 (100.0)	–
	95% CI ^1^	64.6–100.0	81.6–100.0	
	(a) + (b)	5 (71.4)	13 (76.5)	1.000 ^2^
	95% CI ^1^	35.9–91.8	52.7–90.4	
**B. Total Ear**		**Younger Age Group**	**Older Age Group**	***p*-Value**
		***n* = 14**	***n* = 34**	
		**Ears (%)**	**Ears (%)**	
Change in ABR before and after 6 months of treatment	(a) Improved hearing	4 (28.6)	15 (44.1)	
	(b) No change (normal hearing)	1 (7.1)	5 (14.7)	
	(c) No change (same degree of hearing impairment)	7 (50.0)	13 (38.2)	
	(d) Hearing deteriorated	2 (14.3)	1 (2.9)	
	(a) + (b) + (c)	12 (85.7)	33 (97.1)	0.208 ^4^
	95% CI ^3^	69.0–100.0	91.5–100.0	
	(a) + (b)	5 (35.7)	20 (58.8)	0.071 ^4^
	95% CI ^3^	19.0–52.5	40.2–77.5	

ABR, auditory brainstem response; CI, confidence interval. ^1^ Wilson score. ^2^ Fisher’s exact test (both sides). ^3^ Wilson score based on robust standard error considering intraindividual correlation. ^4^ Fisher’s exact test (both sides).

**Table 4:** Comparisons of best ear and total ear hearing using auditory brainstem responses before and after 6 months and 6 weeks of treatment.

**A. Best Ear**			**6 Months** ***n* = 21**	**6 Weeks** ***n* = 21**	***p*** **-Value**
			**Cases (%)**	**Cases (%)**	
Change in ABR before and after treatment	(a) Improved hearing		12 (57.1)	10 (47.6)	
	(b) No change (normal hearing)		4 (19.1)	4 (19.1)	
	(c) No change (same degree of hearing impairment)		5 (23.8)	7 (33.3)	
	(d) Hearing deteriorated		0 (0.0)	0 (0.0)	
	(a) + (b) + (c)		21 (100.0)	21 (100.0)	–
	95% CI ^1^		84.5–100.0	84.5–100.0	
	(a) + (b)		16 (76.2)	14 (66.7)	0.414 ^2^
	95% CI ^1^		54.9–89.4	45.4–82.8	
**B. Total Ear**			**6 Months** ***n* = 42**	**6 Weeks** ***n* = 42**	***p*** **-Value**
			**Ears (%)**	**Ears (%)**	
Change in ABR before and after treatment		(a) Improved hearing	16 (38.1)	14 (33.3)	
		(b) No change (normal hearing)	6 (14.3)	7 (16.7)	
		(c) No change (same degree of hearing impairment)	17 (40.5)	20 (47.6)	
		(d) Hearing deteriorated	3 (7.1)	1 (2.4)	
		(a) + (b) + (c)	39 (92.9)	41 (97.6)	0.273 ^4^
		95% CI ^3^	85.4–100.0	93.1–100.0	
		(a) + (b)	22 (52.4)	21 (50.0)	0.801 ^4^
		95% CI ^3^	36.9–67.8	32.5–67.5	

ABR, auditory brainstem response; CI, confidence interval. ^1^ Wilson score. ^2^ Cochran–Mantel–Haenszel test. ^3^ Wilson score based on robust standard error considering intraindividual correlation. ^4^ Cochran–Mantel–Haenszel test.

## Data Availability

Individual participant data that underlie the results (text, tables, and figures) reported in this article, after deidentification, will be shared following the publication of the article. Requests will be honored from researchers who provide a methodologically sound proposal and execute a Data Use Agreement with the University of Tokyo. Requests should be directed by email to the corresponding author.

## Supplementary Materials

The following supporting information can be downloaded at: https://www.mdpi.com/article/10.3390/jcm11133582/s1, Table S1: inclusion and exclusion criteria of this clinical trial [22], Table S2: summary of the study assessments and procedures [22], Figure S1: summary of the study design [22].

## References

[1] Mestas E. (2016). Congenital cytomegalovirus. Adv. Neonatal Care.

[2] Nagano N., Morioka I. (2020). Congenital cytomegalovirus infection: Epidemiology, prediction, diagnosis, and emerging treatment options for symptomatic infants. Expert Opin. Orphan Drugs.

[3] Koyano S., Inoue N., Oka A., Moriuchi H., Asano K., Ito Y., Yamada H., Yoshikawa T., Suzutani T., Japanese Congenital Cytomegalovirus Study Group (2011). Screening for congenital cytomegalovirus infection using newborn urine samples collected on filter paper: Feasibility and outcomes from a multicentre study. BMJ Open.

[4] Yamada H., Tanimura K., Fukushima S., Fujioka K., Deguchi M., Sasagawa Y., Tairaku S., Funakoshi T., Morioka I. (2020). A cohort study of the universal neonatal urine screening for congenital cytomegalovirus infection. J. Infect. Chemother..

[5] Kimberlin D.W., Lin C.Y., Sánchez P.J., Demmler G.J., Dankner W., Shelton M., Jacobs R.F., Vaudry W., Pass R.F., Kiell J.M. (2003). Effect of ganciclovir therapy on hearing in symptomatic congenital cytomegalovirus disease involving the central nervous system: A randomized, controlled trial. J. Pediatr..

[6] Oliver S.E., Cloud G.A., Sánchez P.J., Demmler G.J., Dankner W., Shelton M., Jacobs R.F., Vaudry W., Pass R.F., Soong S.J. (2009). Neurodevelopmental outcomes following ganciclovir therapy in symptomatic congenital cytomegalovirus infections involving the central nervous system. J. Clin. Virol..

[7] Kimberlin D.W., Jester P.M., Sánchez P.J., Ahmed A., Arav-Boger R., Michaels M.G., Ashouri N., Englund J.A., Estrada B., Jacobs R.F. (2015). Valganciclovir for symptomatic congenital cytomegalovirus disease. N. Engl. J. Med..

[8] Luck S.E., Wieringa J.W., Blázquez-Gamero D., Henneke P., Schuster K., Butler K., Capretti M.G., Cilleruelo M.J., Curtis N., Garofoli F. (2017). Congenital cytomegalovirus: A European expert consensus statement on diagnosis and management. Pediatr. Infect. Dis. J..

[9] Rawlinson W., Boppana S.B., Fowler K.B., Kimberlin D.W., Lazzarotto T., Alain S., Daly K., Doutré S., Gibson L., Giles M.L. (2017). Congenital cytomegalovirus infection in pregnancy and the neonate: Consensus recommendations for prevention, diagnosis, and therapy. Lancet Infect. Dis..

[10] Kawada J., Torii Y., Kawano Y., Suzuki M., Kamiya Y., Kotani T., Kikkawa F., Kimura H., Ito Y. (2015). Viral load in children with congenital cytomegalovirus infection identified on newborn hearing screening. J. Clin. Virol..

[11] Nishida K., Morioka I., Nakamachi Y., Kobayashi Y., Imanishi T., Kawano S., Iwatani S., Koda T., Deguchi M., Tanimura K. (2016). Neurological outcomes in symptomatic congenital cytomegalovirus-infected infants after introduction of newborn urine screening and antiviral treatment. Brain Dev..

[12] Koyano S., Morioka I., Oka A., Moriuchi H., Asano K., Ito Y., Yoshikawa T., Yamada H., Suzutani T., Inoue N. (2018). Congenital cytomegalovirus in Japan: More than 2 year follow up of infected newborns. Pediatr. Int..

[13] Ohyama S., Morioka I., Fukushima S., Yamana K., Nishida K., Iwatani S., Fujioka K., Matsumoto H., Imanishi T., Nakamachi Y. (2019). Efficacy of valganciclovir treatment depends on the severity of hearing dysfunction in symptomatic infants with congenital cytomegalovirus infection. Int. J. Mol. Sci..

[14] Fukushima S., Morioka I., Ohyama S., Nishida K., Iwatani S., Fujioka K., Mandai T., Matsumoto H., Nakamachi Y., Deguchi M. (2019). Prediction of poor neurological development in patients with symptomatic congenital cytomegalovirus diseases after oral valganciclovir treatment. Brain Dev..

[15] Suganuma E., Sakata H., Adachi N., Asanuma S., Furuichi M., Uejima Y., Sato S., Abe T., Matsumoto D., Takahashi R. (2021). Efficacy, safety, and pharmacokinetics of oral valganciclovir in patients with congenital cytomegalovirus infection. J. Infect. Chemother..

[16] Lombardi G., Garofoli F., Villani P., Tizzoni M., Angelini M., Cusato M., Bollani L., De Silvestri A., Regazzi M., Stronati M. (2009). Oral valganciclovir treatment in newborns with symptomatic congenital cytomegalovirus infection. Eur. J. Clin. Microbiol. Infect. Dis..

[17] Bilavsky E., Shahar-Nissan K., Pardo J., Attias J., Amir J. (2016). Hearing outcome of infants with congenital cytomegalovirus and hearing impairment. Arch. Dis. Child..

[18] McCrary H., Sheng X., Greene T., Park A. (2019). Long-term hearing outcomes of children with symptomatic congenital CMV treated with valganciclovir. Int. J. Pediatr. Otorhinolaryngol..

[19] Ziv L., Yacobovich J., Pardo J., Yarden-Bilavsky H., Amir J., Osovsky M., Bilavsky E. (2019). Hematologic adverse events associated with prolonged valganciclovir treatment in congenital cytomegalovirus infection. Pediatr. Infect. Dis. J..

[20] Turriziani Colonna A., Buonsenso D., Pata D., Salerno G., Chieffo D.P., Romeo D.M., Faccia V., Conti G., Molle F., Baldascino A. (2020). Long-term clinical, audiological, visual, neurocognitive and behavioral outcome in children with symptomatic and asymptomatic congenital cytomegalovirus infection treated with valganciclovir. Front. Med..

[21] Dorfman L., Amir J., Attias J., Bilavsky E. (2020). Treatment of congenital cytomegalovirus beyond the neonatal period: An observational study. Eur. J. Pediatr..

[22] Morioka I., Kakei Y., Omori T., Nozu K., Fujioka K., Yoshikawa T., Moriuchi H., Ito Y., Oka A. (2020). Efficacy and safety of valganciclovir in patients with symptomatic congenital cytomegalovirus disease: Study Protocol Clinical Trial (SPIRIT Compliant). Medicine.

[23] Revello M., Gerna G. (2002). Diagnosis and management of human cytomegalovirus infection in the mother, fetus, and newborn infant. Clin. Microbiol. Rev..

