# Supplementary material for: Oral Valganciclovir Therapy in Infants Aged ≤2 Months with Congenital Cytomegalovirus Disease: A Multicenter, Single-Arm, Open-Label Clinical Trial in Japan
Source: J Clin Med. 2022 Jun 21;11(13):3582. doi: 10.3390/jcm11133582 (PMC9267258; doi:10.3390/jcm11133582)
Supplement: Supplementary file 1 [file jcm-11-03582-s001.zip › jcm-1776490-supplementary.pdf]

Supplementary Table S1: Inclusion and exclusion criteria of this clinical trial

	Inclusion criteria		Exclusion criteria
**1**	Confirmation of positive CMV DNA in urine by an in vitro diagnostic test within 21 days after birth	**1**	Infants with bacterial infection requiring antibiotics at the time of study enrollment
**2**	Congenital CMV disease with one or more of the following central nervous system disorders:<br>1) Microcephaly<br>2) Hydrocephalus or ventricular enlargement<br>3) Periventricular calcification<br>4) Cortical hypoplasia or white matter injury<br>5) Retinal choroiditis<br>6) Abnormal auditory brainstem response	**2**	Renal insufficiency (serum creatinine level > 1.5 mg/dL) at the time of study enrollment
**3**	< 60 days of age at informed consent	**3**	Encephalopathy and hydrocephalus owing to other causes
**4**	Gestational age > 32 weeks at birth	**4**	Neutrophil count < 500/mm3 or platelet count < 25,000/mm^3
**5**	Body weight at the time of study enrollment > 1,800 g	**5**	Infants born to women with human immunodeficiency virus (HIV) or infants with HIV
		**6**	Patients deemed inappropriate by a study investigator or sub-investigators

This table is cited from reference #22 (Morioka, I.; Kakei, Y.; Omori., T.; Nozu, K.; Fujioka, K.; Yoshikawa, T.; Moriuchi, H.; Ito, Y.; Oka, A. Efficacy and safety of valganciclovir in patients with symptomatic congenital cytomegalovirus disease: Study Protocol Clinical Trial (SPIRIT Com-pliant). *Medicine* **2020**, *99*, e19765)

Supplementary Table S2: Summary of the study assessments and procedures

		Screening period	Before treatment initiation	Treatment period						After treatment	At the time of drug withdrawal and discontinuation			
			Day 0	Weeks 1, 2, and 3	Week 4	Week 5	Week 6	Weeks 8, 13, 17, and 22	Week 26	Week 30	Drug withdrawal	Discontinuation	4 weeks after discontinuation	6 months after the first administration
Allowable period (days)		-28 to 0		± 2	± 2	± 2	± 2	± 7	± 7				± 7	± 7
Obtaining consent	X													
Obtaining mother's background	X													
Registration		X												
Subject's background		X												
In-hospital /out-hospital		X	X	X	X	X	X	X	X	X	X	X	X	X
Prescription			X	X	X	X	X	X	X					
Viral load in whole blood		X		X	X	X	X	X	X	X		X	X	X
Viral load in urine		X		X	X	X	X	X	X	X		X	X	X
ABR		X					X		X			X		X
Fundus examination		X		when findings are abnormal										
MRI, if necessary CT or US		X							X					
Laboratory test		X	X	X	X	X	X	X	X	X	X	X	X	X
Seizure / paralysis		X			X		X	X	X	X			X	X
Growth evaluation		X			X		X	X	X	X			X	X
Adverse events			X	X	X	X	X	X	X	X	X	X	X	
Blood drug concentration							X							
Discontinuation information				X	X	X	X	X	X	X	X	X		

X indicates that the activity must be performed.

ABR, auditory brainstem response; CMV, cytomegalovirus; CT, computed tomography; MRI, magnetic resonance imaging; US, ultrasonography

This table is cited from reference #22 (Morioka, I.; Kakei, Y.; Omori., T.; Nozu, K.; Fujioka, K.; Yoshikawa, T.; Moriuchi, H.; Ito, Y.; Oka, A. Efficacy and safety of valganciclovir in patients with symptomatic congenital cytomegalovirus disease: Study Protocol Clinical Trial (SPIRIT Com-pliant). *Medicine* **2020**, *99*, e19765)

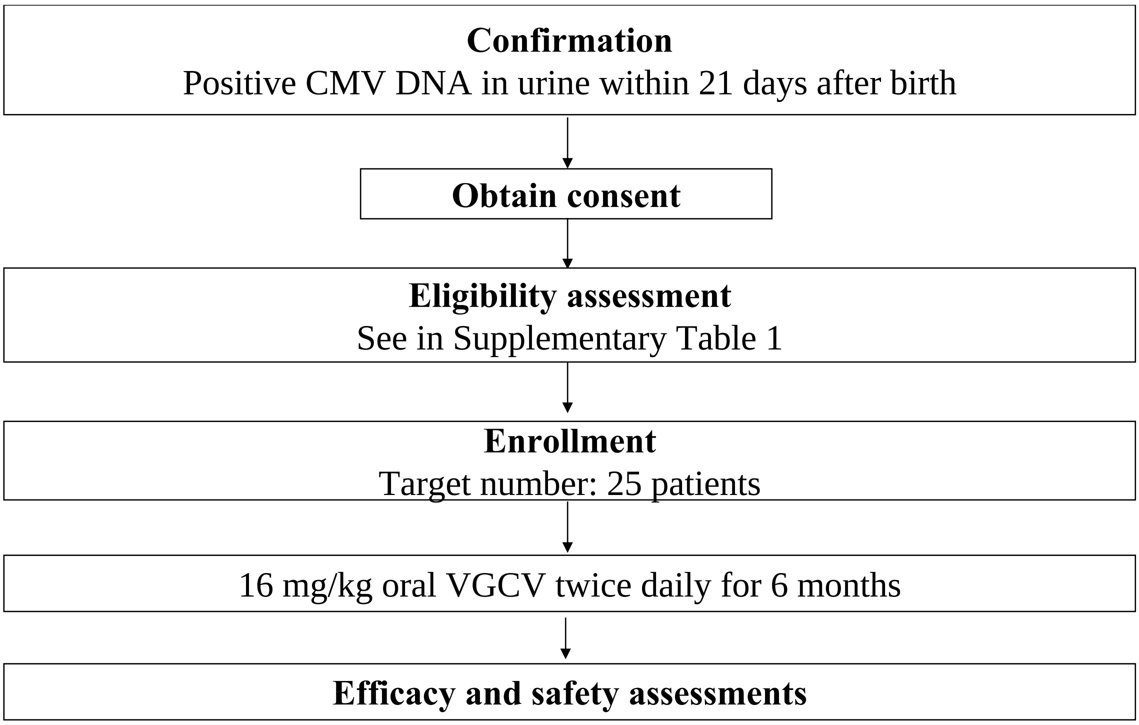

Supplementary Figure S1: Summary of the study design.

CMV, cytomegalovirus; VGCV, valganciclovir

This Figure is cited from reference #22 (Morioka, I.; Kakei, Y.; Omori., T.; Nozu, K.; Fujioka, K.; Yoshikawa, T.; Moriuchi, H.; Ito, Y.; Oka, A. Efficacy and safety of valganciclovir in patients with symptomatic congenital cytomegalovirus disease: Study Protocol Clinical Trial (SPIRIT Com-pliant). *Medicine* **2020**, *99*, e19765) and modified.
